# A Multi-Paradigm Modeling Framework to Simulate Dynamic Reciprocity in a Bioreactor

**DOI:** 10.1371/journal.pone.0059671

**Published:** 2013-03-29

**Authors:** Himanshu Kaul, Zhanfeng Cui, Yiannis Ventikos

**Affiliations:** Institute of Biomedical Engineering and Department of Engineering Science, University of Oxford, Oxford, United Kingdom; Centrum Wiskunde & Informatica (CWI) & Netherlands Institute for Systems Biology, Netherlands

## Abstract

Despite numerous technology advances, bioreactors are still mostly utilized as functional black-boxes where trial and error eventually leads to the desirable cellular outcome. Investigators have applied various computational approaches to understand the impact the internal dynamics of such devices has on overall cell growth, but such models cannot provide a comprehensive perspective regarding the system dynamics, due to limitations inherent to the underlying approaches. In this study, a novel multi-paradigm modeling platform capable of simulating the dynamic bidirectional relationship between cells and their microenvironment is presented. Designing the modeling platform entailed combining and coupling fully an agent-based modeling platform with a transport phenomena computational modeling framework. To demonstrate capability, the platform was used to study the impact of bioreactor parameters on the overall cell population behavior and vice versa. In order to achieve this, virtual bioreactors were constructed and seeded. The virtual cells, guided by a set of rules involving the simulated mass transport inside the bioreactor, as well as cell-related probabilistic parameters, were capable of displaying an array of behaviors such as proliferation, migration, chemotaxis and apoptosis. In this way the platform was shown to capture not only the impact of bioreactor transport processes on cellular behavior but also the influence that cellular activity wields on that very same local mass transport, thereby influencing overall cell growth. The platform was validated by simulating cellular chemotaxis in a virtual direct visualization chamber and comparing the simulation with its experimental analogue. The results presented in this paper are in agreement with published models of similar flavor. The modeling platform can be used as a concept selection tool to optimize bioreactor design specifications.

## Introduction

The diseases of *cellular deficiency* [1] can be only treated if the lost cell population is either regenerated or compensated using autologous substitutes [2], [3]. Given that certain adult human tissues lose their capacity to regenerate [4], they rely exclusively, in case of a critical injury, on functionally similar substitutes [4]–[7]. The principles of tissue engineering can be used to develop such biological substitutes, with remarkably similar properties as those of the host tissues, *in vitro* [4], [6]–[9]. This requires recapitulation of certain key developmental events *ex vivo* thereby necessitating tight control over the artificial growth environment [3], [7], [10]. Bioreactors, which have evolved significantly in both their complexity and functionality over the last two decades, are devices that have been successfully utilized towards this end [2], [3], [10]. Apart from their primary design objective (which is to regulate the cellular microenvironment to support cell viability, promote their 3D organization and provide the cells with spatiotemporally controlled signals) they also offer the user the possibility to seed cells dynamically within 3D matrices, overcome the constraints inherent to static cultures and stimulate the developing constructs physically [3], [10].

Despite the technological advances that have been made in the sector of regenerative medicine and bioreactor technology, there is still a pressing need for safe and clinically efficacious autologous substitutes [3]. Translating regenerative medicine from bench to bed-side would not only require a good product but also robust, controllable and cost-effective manufacturing bioprocesses that are compliant with the evolving regulatory frameworks [3], [11]. Bioreactors serve ideally towards this end as they are the key element for the development of automated, standardized, traceable, cost-effective and safe manufacturing processes for engineered tissues for clinical applications [3].

However, utilized primarily as black boxes, where trial and error eventually leads to the desirable cellular outcome [3], [12], bioreactors have an enormous ground to cover for that eventuality to be realized. Currently, the yields are qualitatively poor and the process of cell growth is often not reproducible. The problem stems from the fact that little is known about the impact of specific bioreactor mass transport characteristics and features on the expansion and growth of cells within the device. Investigators in recent years have begun applying computational tools [12], [13] to study mass transport inside the bioreactor and how that may influence cell dynamics, but this extremely complex interplay has thus far proven elusive.

Analyses based on tackling directly the differential equations governing transport have not only been successful in quantifying mass transport and hydrodynamics inside the bioreactors; their use has been extended to, given certain assumptions, studying cellular dynamics as well [12], [14]. Such models usually either assume absence of neo-tissue within the interconnected pore space in a scaffold or cell attachment only along the surfaces of the scaffold [12]. The differential approach models the cell population, the surrounding extra-cellular framework and nutrients as distributed continua [14]. The matrix in which the cells grow can be treated as a porous medium [14] and one can utilize a wide variety of available computational methods to quantify the distribution of any number of substances being transported and diffusing inside it. Whereas the continuum approach captures the transport phenomena quite accurately, the fact that it investigates biological phenomena at cell *population* level, disregarding entirely the cellular heterogeneity – central to biological function [14], [15] – and the low-level system details [16], hinders detailed analysis of cellular dynamics [11], [17], [18].

In order to understand the impact of cell level behavior on the overall cell population discrete models can be employed [14]–[16], [18]. The cellular automata approach has been used extensively to trace the microscopic details of cellular dynamics more directly and accurately by attributing a set of evolution/transition rules to the computational grids that can represent biological entities such as the cell or the physical microenvironment [14], [19]. The models that have been tried using this approach usually assume a constant supply of nutrients, which is not fully reflective of the actual conditions even under carefully designed experiments [15], [20]. Furthermore, the discrete models available in the literature, despite capturing processes such as contact inhibition, persistent random walk and cell division with marked accuracy, do not consider the impact of chemotaxis and apoptosis on the overall growth dynamics of a cellular colony [15], [20]. More recently, hybrid models, which are a combination of the continuum and discrete approaches, have been utilized to study the impact of transport phenomena on cellular dynamics [14], [16], [21], [22]. Despite being a significant advancement over both the continuum and discrete approach, most of the limitations of the cellular automata models apply to the hybrid models as well. Additionally, the fact that these models are computation- and time- consuming makes it difficult for them to be considered for three-dimensional systems.

Before exploring the relevant hybrid models it needs stating that the hybrid approach itself is not novel and has been applied to simulate a variety of non-biological phenomena. Examples include coupled finite element-flux corrected transport method/finite volume approach to predict electrostatic fields, electrohydrodynamic flow, particle charging and turbulent motion, and their mutual interaction in 3D models of a single wire-plate electrostatic precipitator [23]; finite-element/finite-volume approach to model flow and transport in heterogeneous porous media [24]; computational-fluid-dynamics (CFD)/agent-based modeling (ABM) approach to simulate a gas turbine engine [25]; finite-difference/finite-element approach to model temperature increase in biological vascularized tissues produced by radio-frequency exposure [26]; and discrete element method/compartment modeling to analyze granular mixing [27] amongst others. Furthermore, attempts have been made to study gas-liquid flow in bubble column reactors [28] and gas-liquid-solid three phase flow using a Lagrangian-Eulerian approach [29], [30].

Chung et al (2006) [5] developed a mathematical model to explain tissue growth inside a scaffold by treating the cell-scaffold construct as a porous medium, also incorporating cell diffusion to account for cell random walks. Galban and Locke (1999a, 1999b) [31], [32] adopted a similar approach and utilized species continuity equations and the volume average method to model *in vitro* growth of cartilage tissues. Both these modeling efforts produced interesting results and valuable insight – within the limitations of continuum models of course. Lemon and King (2006) [33] utilized a multiphase model to capture the growth of biological tissue inside a rigid scaffold. The model, based on the mixture theory where each tissue component – cells, water and a solid scaffold material – was treated as a continuum on the macroscale, accounted for cell division as well as apoptosis. Although it dealt very elegantly with the mechanical aspects of the system, necrosis was not considered in the model. Moreover, mitosis was considered to be proportional to the volume fraction of nutrients, cells and water; whereas apoptosis was considered to be proportional to the volume fraction of cells – thereby disregarding the dependence of such behavior on cellular and spatial heterogeneity. Similarly, Flaibani et al (2010) [34] modeled the spatiotemporal evolution of cell heterogeneity in a porous scaffold by solving the relevant PDEs, (discretised using the finite volume approach). The model considered perfusion conditions. These models can capture the population level behavior quite adequately, yet involve assumptions that lead to ignoring of important behavior such as cell migration, apoptosis, necrosis, chemotaxis, variations in the spatiotemporal microenvironment. Thus, a comprehensive picture of the synergistic dynamic interplay that exists in biological as well as tissue engineering systems remains a challenge.

On the other hand, Cheng et al (2006) [15] used the discrete approach to model the dynamic process of tissue growth in a 3D environment. Their model was an improvement over a 2D model developed by Lee et al (1995) [20]. The model considered a population of cells executing persistent random walks on the computational grids, cell-cell collisions and proliferation until confluence. The model assumed constant nutrient and growth factor concentration in space and time and did not consider cell death (apoptosis or necrosis) and chemotaxis. In a more recent model, Cheng et al (2009) [16] utilized the continuum-discrete approach to model the complex interplay that exists between cell populations and mass transport dynamics. Cell interactions were modeled using the discrete CA approach whereas diffusion and consumption of nutrients were based on a transient PDE approach. The dependence of cell division and cell migration on nutrient concentration, which is not to be confused with chemotaxis, was also accounted for. As migration speed was proportional to nutrient concentration, lower nutrient concentration meant lower migration speed. Although the latest model presented by Cheng et al (2009) [16] remains one of the most complete in the literature, it too did not consider chemotaxis and necrosis. Galbusera et al (2008) [22] adopted a similar strategy to create a software framework for computational modeling of tissue engineering experiments. Cell population in this framework is modeled using the ‘discrete cells in a continuum space’ (Galbusera et al., 2008) approach [22]. The finite element approach was used to model the cell environment. The group presents a 3D microscopic model but only a 2D macroscopic model. Michaelis-Menten kinetics were used to calculate oxygen consumption by the cells (which makes oxygen consumption a population behavior). Furthermore, the model considers necrosis, due to lack of oxygen, occurring when the oxygen concentration falls to less than 50% of the initial value. The model did not consider chemotaxis.

Despite their focus on microorganisms, hybrid models developed by Lapin et al deserve a mention due to the ease of extension of the models to animal cells. Lapin et al modeled microorganism population behavior in bioreactors by opting for an individual-based approach [35]–[37] whereby the dynamic behavior of the system as a whole can be traced to the behavior of individual organisms. Their initial model [35], [37] focused on simulating temporal and spatial behavior of a population of oscillating yeast cells based on glucose concentration fields in a bioreactor. In order to achieve this, computational fluid dynamics (CFD) – modeling the turbulent flow fields in the bioreactor – was coupled with Eulerian-Lagrangian representation of the system, where the extracellular environment was based on the Euler approach and the distributed biophase was characterized by a discrete cell ensemble (Lagrange) approach. The model considers cell migration by superimposing random movement due to turbulent dispersion on the convective flow. The cell in this instance, however, does not mean a ‘real’ living cell, rather a computational element that represents a large collective of real cells. In its advanced form [36], [37] the model was extended to simulate E. coli population dynamics in a stirred-tank bioreactor with non-ideal mixing. In particular, Lapin et al modeled glucose uptake by the bacteria, which depends on a combination of the extracellular glucose as well as intracellular metabolite concentrations. The investigators observed distinct differences in cell viability at various scales of operation. The novelty of the model lies in its strategy to trace population behavior by considering the individual cell response as a result of key reactions of the central metabolism, which we feel is a more mature, if computationally expensive, way of approaching biological complexity. Certain assumptions of this model are worth highlighting here: firstly, the Lagrangian representation of the model is pseudo-discrete. Each computational element represents a population of physical cells. It can be argued that this makes the simulation computationally economical but has the disadvantage of ignoring various individual-level details. Furthermore, like others previously discussed, the models do not consider proliferation, chemotaxis, or apoptosis – features particularly important in tissue engineering bioreactors (the focus of our work).

A modeling approach that has been gaining interest amongst biologists and mathematicians alike is the agent-based modeling (ABM). Drawing on different fields such as computer science, artificial intelligence, complex systems, and the social sciences [18], [19], [38]; ABM belongs to a class of discrete mathematical approaches in which a system is modeled as a collection of autonomous decision making entities that possess the capacity to detect local information and act at each of several discrete *time steps* based on a set of logical and/or mathematical rules attributed to them [19], [39]. Although quite similar in flavor to the cellular automata (CA) approach, ABM differs from CA in that ABM employs mobile agents, is characterized by asynchronous agent behavior – i.e. allowing agents to update their states independently of each other – and allows the user to incorporate stochastic elements in the rule-set attributed to the agents [19]. Furthermore, the CA approach, which can be described as a fixed grid of interacting finite-state machines, lacks internal memory, which leads to a combinatorial explosion of stages when considering even trivial communication [17]. As a result, when it comes down to representing complex systems, the agent-based approach appears to offer certain advantages over the cellular automata approach.

A design tool capable of predicting the impact a bioreactor’s design specifications, such as its flow-rate, inlet/outlet position, geometry, and a given cell’s biological properties, such as its nutrient consumption or metabolic rate, can have on the growth (and differentiation) dynamics of the overall cell population will therefore not only be immensely helpful in optimizing bioreactor design and construction, but may help uncover the governing dynamics that regulate development. In this study a multi-paradigm ‘transport-agent’ model, capable of predicting, based on a set of logical, algebraic, stochastic and differential rules, the impact of bioreactor mass transport and hydrodynamics on the growth dynamics of cells in a virtual bioreactor is presented. The novelty of the platform is that in addition to capturing cell dynamics as a result of interactions between individual cells (a feat previously achieved by published CA and agent-based models), it also considers the impact that local transport has on the cells and how the cells might be able to indirectly alter their local environment due to behavior like cell division, cell aggregation or extra-cellular matrix synthesis or digestion. The platform can therefore capture *dynamic reciprocity* [40], [41]; an emergent phenomenon.

To achieve this, we have pursued the tight coupling of two mature modeling platforms; first, the Flexible Large-scale Agent Modeling Framework (FLAME) [17], [42]–[45], an agent-based system, with a computational multi-physics transport phenomena platform (CFD-ACE+, ESI Group, Paris, France). FLAME captures the rules that govern cell growth and proliferation whereas CFD-ACE+ is employed to simulate bioreactor hydrodynamics, mass transport processes and other biomechanical effects (for example, shear or strain triggered cellular responses). The platform considers cellular behavior in 3D. Through the platform we wanted to test the hypothesis that bioreactor geometries, bioreactor variables and initial conditions are crucial to cell development and that the integrated framework could be used to capture that and optimize bioreactor design. In this paper, various bioreactor variables are tested virtually. The results of the *in virtuo* experiment deploying the integrated model are presented and discussed. The bioreactor models considered were relatively simple, although the platform has the capability to deal with geometries, perfusion/stimuli characteristics and cellular populations of arbitrary complexity.

## Methods

Quantifying cell population dynamics as well as the biophysicochemical microenvironment are the important aspects of modeling tissue engineering systems [22]. As discussed above, the impact of spatial and cell-population heterogeneity can be best modeled using the discrete approach whereas the continuum approach remains the most accurate way of capturing the bulk phenomena. The modeling platform was therefore composed of two integrated and communicating elements that can simulate the various biological processes which work synergistically to produce behavior of staggering complexity. A brief description of each of the two components is therefore essential.

### 2.1 Agent-Based Modelling

When the number of individuals to be modeled in a process is relatively small (roughly 10^5^–10^6^), emergent phenomena are the primary interest and spatial considerations are important (as individual entities can be localized in space), an agent-based approach can be utilized [43]. As defined by Wooldridge [46], “*an agent is an encapsulated computer system that is situated in some environment and that is capable of flexible, autonomous action in that environment in order to meet its design objectives*”. Therefore, by definition, an agent possesses well defined boundaries, has the ability to sense its environment and act on its environment, can control its internal state as well as behavior, have particular goals to achieve, can act in the anticipation of future goals, and respond in timely fashion to changes that affect its environment [46]: features that in principle make an agent very similar to a cell.

The agent-based approach decomposes the problem in terms of autonomous entities. These autonomous entities engage in flexible, high-level interactions, a feature that attributes to the system multiple loci of control. Decision-making is therefore limited to the agents’ actual situation as opposed to some external entity’s perception of this situation [46]. The fact that agent interactions are flexible allows the user to attribute to the components the ability to make decisions about the nature and scope of their interactions at run-time, thereby bypassing the need to specify every possible inter-agent link (an impossibility given the nature of any biological system’s complexity) [46].

The agent-based paradigm possesses structures that can represent and manage organizational relationships, such as roles, norms and social laws [46]. Furthermore, the presence of interaction protocols (to form new groupings and disband unwanted ones) [46] coupled with the ease with which collectives, such as teams, could be modeled enables the agent-oriented mind-set to provide suitable abstractions [46] necessary to model complex, especially biological, systems. And finally, the fact that ABM can conduct an organizational updating [46] during run time (in case an agent is destroyed) makes the agent-based philosophy more suitable to address the dynamic nature of biological systems.

Chavali et al (2008) [19] listed desirable framework capabilities that are important to address key challenges in immunology. They can be extended to frameworks that are being developed to capture cellular behavior. Such frameworks must simulate non-linear and dynamic behavior, cell-cell and cell-environment interactions and cell population behavior as a function of population heterogeneity; attribute the cells with features such as memory (to keep track of various prior interactions) and adaptability (based on the external environment); and permit visualization of the resulting phenomena that emerges from the combined interactions between the cells considered in the model [19]. An agent-based method, in particular the Flexible Large-scale Agent-based Modeling Environment (FLAME), provides the investigator the ability to do precisely that.

Moreover, FLAME allows simulation of large numbers of agents to be run on parallel computers [17], [42]. The platform was developed at the University of Sheffield for the *Epitheliome* project and has been used to model the emergent behavior of biological as well as economic systems [18], [42]. The FLAME framework, which enables creation of agent-based models that can be run on high-performance computers, is based on the logical communicating extended finite state machine (X-machine) theory [17], [47]. Agents are modeled as communicating stream X-machines, an attribute that allows them to interact with each other. This modeling mechanism provides a sensible way of dealing with problems associated with state explosion, which afflict many efforts at modeling complex biological systems [17], [43], [45]. Furthermore, being inherently hierarchical, an X-machine is able to link different modeling paradigms; an attribute that is critical to the success of this platform [43]. For more information on FLAME, the interested reader is directed to http://www.flame.ac.uk/.

### 2.2 Transport Phenomena

The hydrodynamics inside the bioreactor as well as the mass transport were quantified by solving the governing transport equations (i.e., conservation of mass, momentum and species) using the finite-volume method. In this methodology, the computational domain is divided into a set of control volumes (CVs) by means of a grid. The finite volume method, by using the integral form of the general transport equation, preserves its conservative nature. The generalized transport equation for a conserved quantity Φ is shown below as equation 1. This equation, by appropriately assigning parameters and source terms, effectively accounts for the conservation of mass, momentum, species and reaction of species. In the equation *ρ* is the fluid density and *U* the velocity vector. On the left hand side of the equation: the first term accounts for the transient nature of the process, the second for convection and the third for diffusive processes. The term on the right hand of the equation, a generalized source term, accounts for variable-specific mechanisms; such as the pressure gradient in the momentum equation manifestation of the general transport equation, or the chemical reaction as far as the species conservative equation is concerned, or the secretion or consumption of a molecule by the cells, when continuity is considered.
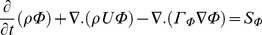
(1)


Equation 1 was solved in its full transient form. As a suitable abstraction, oxygen was assumed to be the limiting scalar, although the platform can consider multiple scalars and, if necessary, can capture any reactions that may exist between these scalars. Consumption of oxygen was modeled by representing the cells, or more appropriately the agents, as proliferating sinks – covered by the term on the right in equation 1. It must be noted here that this equation is easily extended, in a Darcian sense, to account for porous media, by incorporating local porosity and permeability terms (i.e. local void fractions and resistances) at every computational cell.

Converting the integral of (1) over the CV into a surface integral yields equation (2), where S represents any of the faces on the CV, whereas **n**_S_ is the unit vector normal to that surface. The convective and diffusive terms are determined using suitable second order accurate interpolation schemes [48].
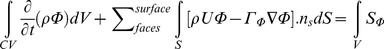
(2)


Pressure and velocity fields were coupled using the SIMPLEC algorithm [49] and an *algebraic multigrid* (AGM) solver was employed [50] as the iterative equation solver. The AGM solver uses a hierarchy of grids, from fine to coarse, and back to fine, to solve the resulting set of pseudo-linear equations: After obtaining the residual on the fine grid, iterations are performed on the coarse grid to obtain corrections (imposing fine-grid residual as the source term). The AGM solver works by interpolating the corrections to the fine-grid and updating the fine grid solution, and repeating the entire procedure until the residual is reduced to the desired level. This way, errors of multiple wavelengths are improved upon simultaneously.

**Figure 1:**
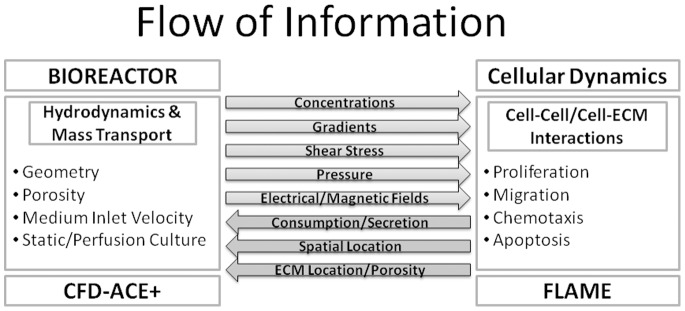
Flow of Information in the modeling framework. The figure shows the communication between the transport-phenomena and agent-based modules that is at the heart of the modeling platform. Information relevant to bioreactor hydrodynamics and mass transport is communicated from the transport-phenomena module to the agent-based module where cells, modeled as agents, detect the local concentrations (and other continuum variables) and act based on the rules attributed to them. The cellular information is then relayed back to the transport-phenomena module to complete the circuit.

The numerical procedures described above were implemented in CFD-ACE+ in this study. This is a multi-physics proprietary computational tool that allows easily the interfacing with external modules, thus incorporating additional physics (for example, the effect of electrical or magnetic fields, temperature or deformable substrates on cells). Integrating FLAME with CFD-ACE+ provides an efficient multi-paradigm modeling framework that was used to set up a multi-scale model displaying cellular dynamics inside a virtual bioreactor. Figure 1 shows the exchange of information between the agent-based model and the transport model that is at the heart of the platform presented in this paper.

### 2.3 Model Features

#### 2.3.1 oxygen transport and consumption

**Figure 2:**
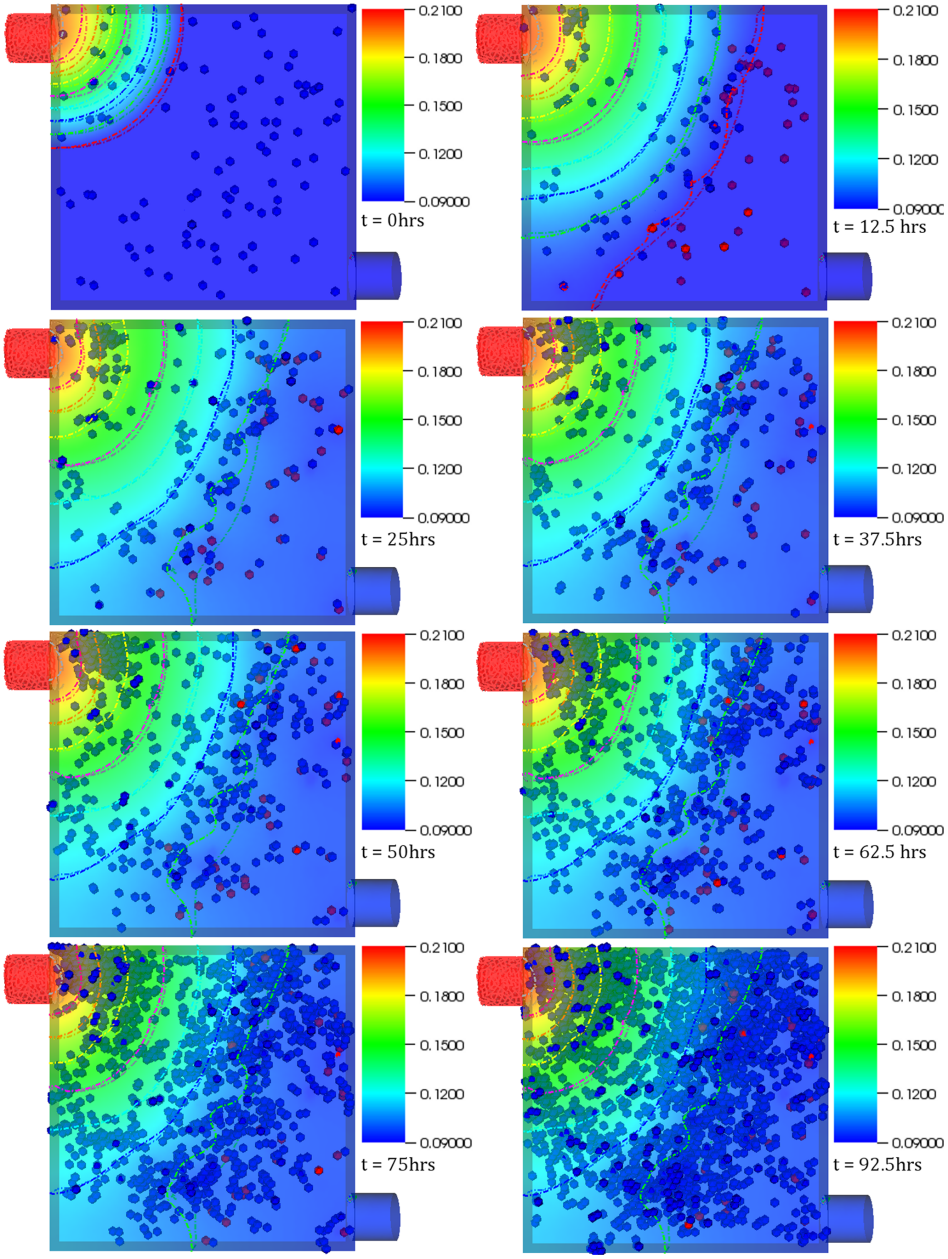
Case 1 results. Temporal evolution of cell population and nutrient concentration inside a 3D scaffold bioreactor (geometry A) with a medium inlet velocity of 0.001 m/s. The top-left port on the bioreactor serves as the inlet whereas the bottom-right port serves as the outlet. The final frame captures cell distribution at the end of 4 (physical) days – the time interval between snapshots (left to right) is 12.5 hours. The initial cell density was 100.

**Figure 3:**
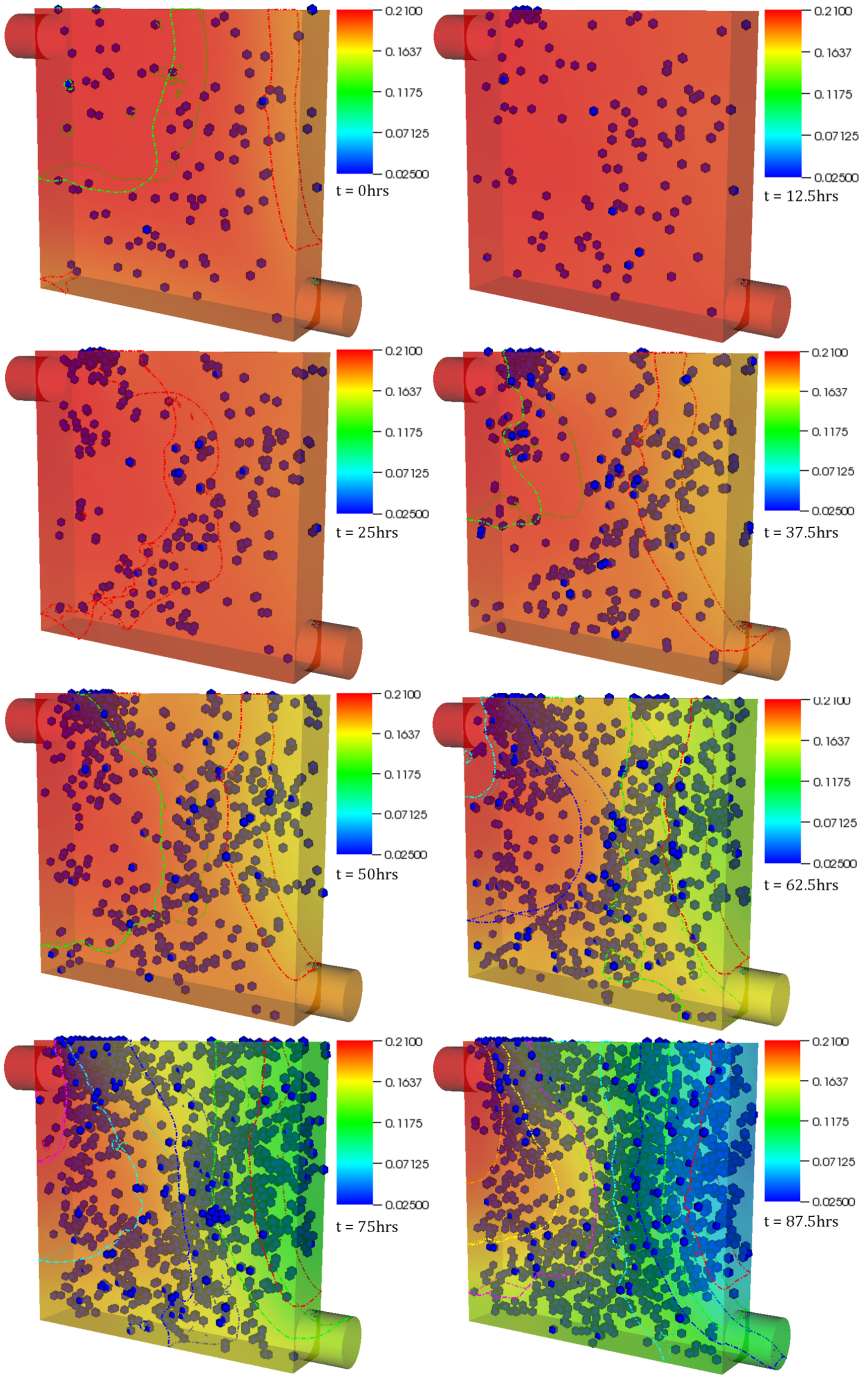
Case 2 results. Temporal evolution of cell population and nutrient concentration inside a bioreactor (geometry A) with a medium inlet velocity of 0.01 m/s. The final frame captures cell distribution at the end of 4 (physical) days – the time interval between snapshots (left to right) is 12.5 hours. The initial cell density was 100. The concentration contours can be observed to change continuously throughout the simulation. This is in contrast with physical systems with no cells inside where such behavior would not be possible after the flow becomes stationary beyond initial transients. This demonstrates the platform’s ability capture dynamic reciprocity.

The cells were assumed to be seeded in a porous scaffold inside a bioreactor and were supported by the influx of oxygenated medium. Two virtual bioreactors, enclosing a porous scaffold, of different geometries but same volume were constructed. The two bioreactor geometries can be seen in Figures 2, 3 (geometry A) and 4, 5 (geometry B), and the dimensions of the bioreactors are listed in Table 1. Bioreactor construction was followed by a grid independence analysis, which was conducted on one of the bioreactors (geometry A). The bioreactor was assigned structured (50,000; 100,000; 200,000 and 400,000 elements) as well as unstructured (100,000 and 400,000 elements) grids. The results indicated no appreciable difference between a bioreactor with a 100,000 element structured grid and a bioreactor with a 400,000 element unstructured grid. As a result, to strike a balance between result accuracy and computational time, the bioreactors were solved using 100,000 elements structured grids. Furthermore, the scaffolds were assigned constant isotropic porosity and permeability (75% and 10^−10^ m^2^ respectively) and tested for medium inlet velocities of 0.01 m/s and 0.001 m/s. Please refer to Table 2 for a description of different test cases.

**Table 1:** Bioreactor Variables.

Bioreactor Variables		
Geometries	2	
Scalar	Oxygen	
Initial Concentration^14^	0.21 mol m^−3^	
Scalar Diffusivity in the Media^14^	10^−5^ m^2^/h	
Scaffold Porosity	75%	
Scaffold Permeability	10^−10^ m^2^	
Medium Density	1000 kg/m^3^	
Medium Viscosity	0.001 kg/m-s	
Medium Inlet Velocity	0.001 m/s, 0.01 m/s	
Bioreactor Dimensions	Length	1 mm
	Width	1 mm
	Depth	0.2 mm

**Figure 4:**
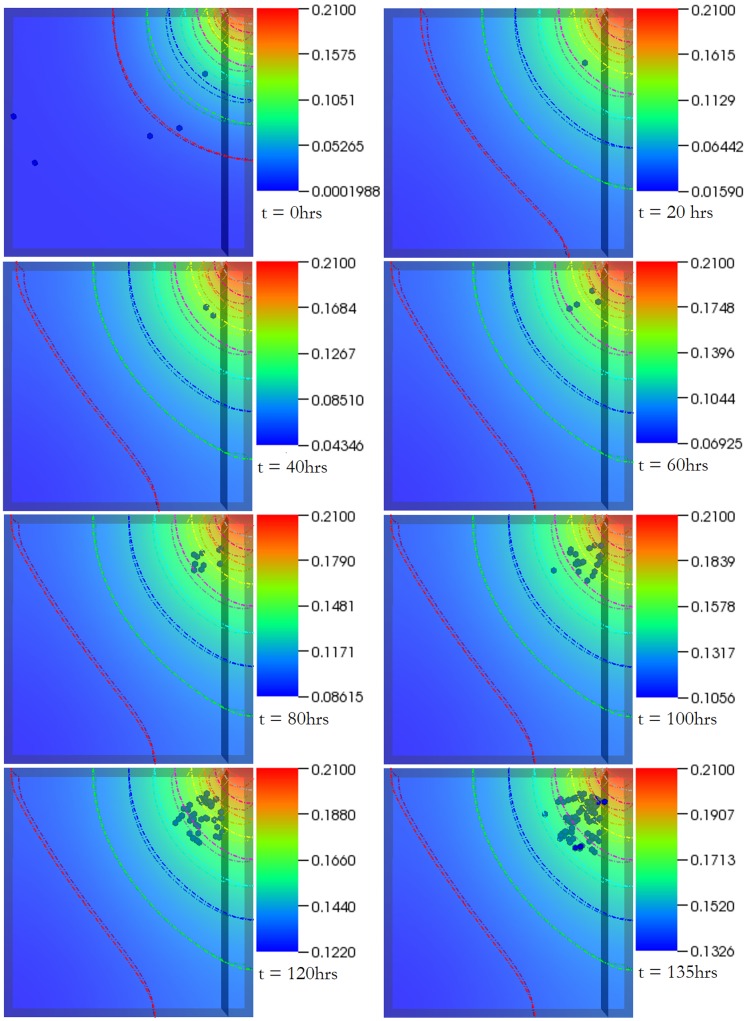
Case 3 results. The figure shows temporal evolution of cell population and nutrient concentration inside a bioreactor (geometry B) with a medium inlet velocity of 0.001 m/s. The top right end of the bioreactor serves as the inlet whereas the entire left as well as bottom ends of the bioreactor serve as outlet. The initial cell density was 5. The time interval between snapshots (left to right) is 20 hours. The final frame captures cell distribution at the end of 6 (physical) days.

The table lists various volume, boundary, and initial conditions applied to compute mass transport inside the bioreactors. The dynamic relationship between cell proliferation and mass transport of oxygen was investigated in two bioreactors of same volume but different geometries (shown in figures 2 and 4). Oxygenated medium was introduced at two different velocities: 0.001 m/s and 0.01 m/s.

**Table 2:** Test Cases.

Case	Bioreactor Geometry	Initial Cell Density	Medium Inlet Velocity (m/s)	Flow Rate (L/s)	Relevant Figures/Videos	
Case 1	A	100	0.001	0.02	Fig 2/S2	
Case 2	A	100	0.01	0.2	Fig 3/S3	
Case 3	B	5	0.001	0.002	Fig 4/Fig 5/S4	
Case 4	B	5	0.01	0.02	Fig 5/S5	

The table lists various test cases simulated for the purposes of this investigation and relevant parameters, such as medium flow rate, and relevant figures and supplementary material.

Concentration gradient of oxygen is known to affect tissue-growth rate in bio-artificial scaffolds [16]. Therefore, the model was designed to study cell growth in a continuous medium perfusion system with oxygen being the limiting nutrient. After the bioreactor-scaffold complex was suffused with virtual cells, oxygenated medium was pumped in at the velocities (and corresponding flow rates) listed in Table 2. Oxygen transport inside the bioreactor occurred by convective as well as diffusive processes. The diffusivity of oxygen in the medium was taken as 10^−5^ m^2^/hr [14]. The medium supplied to the bioreactors was assumed to be carrying oxygen at a concentration of 0.21 mol/m^3^ [14]. Oxygen consumption was modeled using cells as proliferating and migrating non-zero sinks consuming oxygen at 3.39 mol kg m^−3^ s^−1^ [14]. In the agent-based component this amounts to oxygen consumption by each cell at a rate of 12.2 mol m^−3 ^hr^−1^.

Oxygen (or any other substance) consumption (or secretion), modeled as an individual-level event, was accounted by the source term represented as S_Φ_ in (1). The equation was implemented as migrating non-zero sinks. Generally speaking, the source term can be represented by equation 3, which displays the dependence of S_Φ_ on existing scalar concentration.
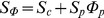
(3)


Equation 3 involves a constant as well as a linear dependence of the source term on scalar concentration. In cases where the relationship is non-linear, it must be linearized [51]. Equation 3 was applied to control volumes with cells in them (as derived from the agent-based module of the platform) and with appropriate summations in the case of multiple cells within a single control volume.

Oxygen concentration in this model is assumed to vary based on the bioreactor hydrodynamics, mass transport and cell proliferation. Dirichlet and Neumann boundary conditions were applied as needed. Oxygen transport and consumption, in sync with cellular proliferation and migration, was calculated for periods of four to six days depending on the case and the fate of the cells in the virtual bioreactors.

#### 2.3.2 Cell population dynamics

The platform is designed to incorporate a variety of behaviors displayed by cells: migration, proliferation, differentiation, chemotaxis, apoptosis, necrosis and other processes as needed. The agent-based component considers each virtual cell to be an agent governed by a set of logic rules that is capable of displaying migration, proliferation, chemotaxis and apoptosis. Differentiation was not considered in the cases tested in this paper. The biological rules governing the virtual cells, listed in Table 3, are controlled by constants, for example cell cycle, as well as variables – which in turn emerge from the transport phenomena component – such as oxygen concentration gradients. The cells were assumed to be non-deformable spheres of radius 10 µm each and capable of consuming oxygen at a rate of 12.2 mol m^−3^hr^−1^cell^−1^ when available. Initial cell placement inside the bioreactor-scaffold complex was random.

**Table 3:** Cellular Variables and Rules.

Cellular Variables	
Persistence Time^14−16, 20, 53^	2 hours
Post Collision, pre-Division Stationary Phase^20^	1 hour
Cell Cycle & Division Probability of Cell Population^53^	18 hours (64%)
	24 hours (32%)
	32 hours (4%)
Maximum Random Speed	10 µm/hr
Cell speed during Chemotaxis	20 µm/hr
Oxygen Consumption Rate^14^	3.39 mol kg m^−3^ s^−1^
Chemotaxis Ensues at Scalar Concentration^52^	0.0672 mol m^−3^
Hypoxia-induced Apoptosis	15 hours
Cell Density in Bioreactors	100, 5

The table lists parameters pertinent to the cells as well as rules the cells followed during the computation. For example, under normoxic condition cells displayed persistent random walk changing direction once every two hours. Cells would only stop if it is in contact with another cell or about to undergo mitosis. Furthermore, chemotaxis ensues if a cell experiences local oxygen concentration of less than 0.0672 mol m^−3^. Failure to move to a normoxic region within 15 hours since the inception of chemotaxis leads to hypoxia-induced apoptosis.

The cells could migrate by choosing either a persistent random walk or chemotaxis. The cells displayed persistent random walk [15], [16], [20] if the local oxygen concentration >0.0672 mol/m^3^. If, however, the local oxygen concentration dropped below 0.0672 mol/m^3^, the cells began to display chemotaxis in a bid to move to an oxygen-rich region. This value is equal to 0.3% of the initial oxygen concentration, and was decided upon after reviewing the work of Liu et al (2007) [52] who reported induction of hypoxia at oxygen concentration of 0.3% in HCT 116 colon carcinoma cells. The cells were assumed to divide until confluence or until the point where there was not enough oxygen available to them. Confluence, or the point where the bioreactor is completely filled with cells, was achieved when each cell was bonded to at least four other cells. The cells divided based on a division probability assigned to them: 64% of the cells divided by eighteen hours, 32% by twenty four hours and the remaining 4% by thirty hours [53]. The daughter cell is positioned at a random orientation relating to the coordinates of the parent cell, and in the immediate vicinity of the parent cell.

Another advancement this platform has to offer is that it takes into account cell apoptosis that may occur due to cells experiencing hypoxia in oxygen deficient areas created in the bioreactor due to cell growth or other factors such as low medium inlet rate or deficient mixing. If the local oxygen concentration dropped below 0.0672 mol/m^3^, the cells began to express apoptotic proteins. If a cell stayed under the hypoxic condition for more than 15 hours, it died of hypoxia-induced apoptosis. The cells were assumed to exert a repulsive force on each other in case of contact, which was taken from a model developed by Tao et al (2007) [44]. Although the nature of mechanical forces that cells exert on each other is quite complex, the physical forces were resolved using constants instead of variables as our primary objective was to display the platform’s capability to handle such occurrences and to accommodate any such sub-model when available. Please refer to Table 3 for relevant parameters.

#### 2.3.3 Cell migration

The extracellular environment and cell type affect and dynamically modulate [16] a cell’s speed and its persistent time [15], with prostate cancer cells displaying speeds of 8–15 µm/hr in 3D collagen matrices and melanoma cells 20–40 µm/hr in 3D collagen matrices modified with RGD proteins [15], [54], [55]. The scaffold in our case was assumed to have no restraining effect on cell migration. Therefore, despite their presence in a porous scaffold, the cells could move freely in all (three-dimensional) directions. A migration speed was assigned to each cell. As long as the cell displayed persistent random walk, it could acquire a maximum speed of 10 µm/hr. The cell continued moving in a particular direction for two hours after which, based on the availability of space, the cell assumed a new randomly chosen direction, in agreement with [14]–[16], [20], [53]. If, while migrating, a cell came in contact with another cell or the bioreactor boundary, it stopped for an hour, in agreement with [14]–[16], [20], [53], before changing its direction and continuing migrating in a randomly chosen direction. The cells stopped moving prior to dividing and, along with the daughter cell, remained at rest for about an hour after division [20]. While displaying chemotaxis, the speed and direction attributed to the cells were based on the local concentration gradients. Under chemotaxis the cells were assumed to display a set migration speed of 20 µm/hr. If, while performing chemotaxis, a cell ended up in a region rich in oxygen, it went back to displaying the persistent random walk; if not, then the cell moved under the influence of the concentration gradient until it either ended up in an oxygen rich area or it died.

Apoptotic trigger was initiated if the local oxygen concentration dropped below 0.0672 mol/m^3^. If a cell remained in an oxygen-deficient region for more than 7 hours, it changed its state (and its color in the visualization platform used to analyze the results of the simulations) indicating that the apoptotic mechanism, physically represented by formation of apoptotic proteins in the cells that lead to cell death, has been triggered. If the cell, in chemotaxis mode at this point, was successfully able to relocate to an oxygen rich region it survived; otherwise if it remained in a hypoxic environment for more than 15 hours since the start of the apoptotic mechanism, it died. The time advancement of the system was organized around computing cycles, usually referred to as *iterations* in agent-based modeling. Since this term has a different meaning in transport phenomena implicit solvers, we shall refrain from using the term – it suffices to say that each computing cycle or iteration was set to 15 minutes and we have found this to be a value that captures the fine features of the system, while leading to reasonable computational time requirements. Table 4 summarizes the rules used in the study.

**Table 4:** Rules.

Behavior	Rule
Proliferation	Cell proliferation occurs until confluence as per assigned probabilities (Table 2)
Migration	Migration occurs either via persistent random walk (under normoxic conditions) or chemotaxis (under hypoxic conditions)
	If local oxygen concentration >0.0672 mol/m^3^, cells display persistent random walk
	Cell speed during persistent random walk = 10 µm/hr
	If local oxygen concentration <0.0672 mol/m^3^, chemotaxis begins
	Chemotaxis speed = 20 µm/hr
Apoptosis	If a cell experiences hypoxia for more than 15 hours, it dies of hypoxia-induced apoptosis
	If, while performing chemotaxis, a cell ends up in a region rich in oxygen, it goes back to display persistent random walk

The table summarizes the rules used to simulate cellular dynamics.

### 2.4 Experimental Validation

The ability to capture cellular chemotaxis is a novel feature of the platform. An understanding of the detailed mechanism of chemotaxis finds relevance in, among other sectors, cancer research [56]–[58] and cancer drug design [59]. Typically, the assays utilized to investigate chemotaxis are based on the *two-well* design. Briefly, two wells – one containing a control or buffer substance, and the other the chemoattractant – are connected to each other. Cells are seeded between the wells where they can sense the developing gradient and display an appropriate migration response [60]. Direct visualization assays allow the user to observe cell migration in real-time with the aid of time-lapse microscopy, and are considered the gold standard assay for investigating chemotaxis [60], [61]. Therefore, in order to experimentally validate the platform we simulated cellular chemotaxis in a direct visualization chamber. To achieve this a virtual analogue of the *Insall Chamber* [60] was created. The Insall chamber is a direct visualization chamber developed by *Muinonen-Martin et al* [60] to study chemotaxis using high numerical aperture (NA) oil immersion lenses, which was not possible with other visualization chambers. The Insall chamber consists of an inside well containing the control and an outside well (enclosing the inner well) containing the chemoattractant. The investigators analyzed chemotaxis of MV3 melanoma cells based on linear concentration gradients of Fetal Bovine Serum (FBS). Details regarding the chamber and the experiment can be found here [60].

**Figure 6:**
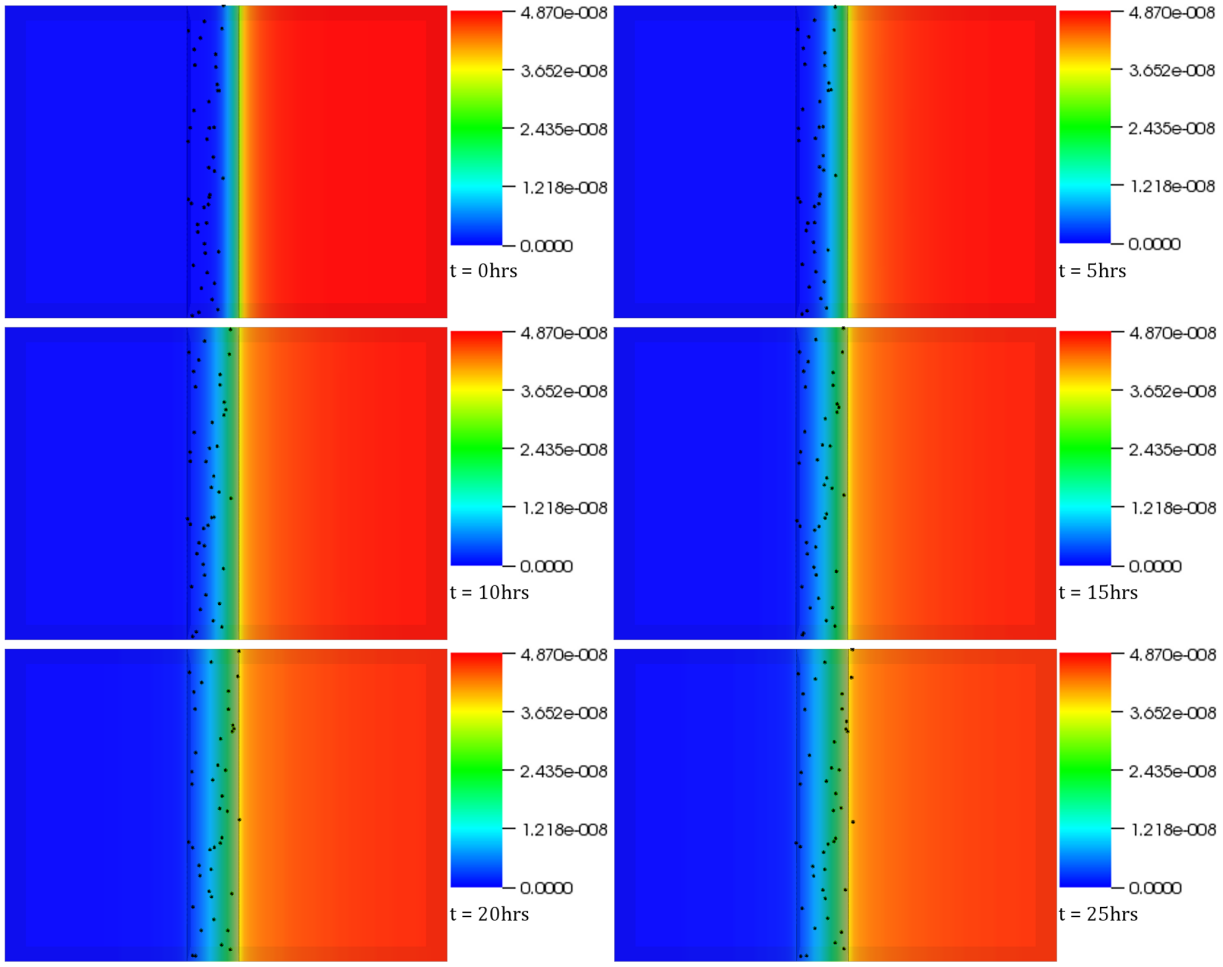
Experimental Validation of the platform. The figure shows migration response of MV3 melanoma cells based on FBS concentration gradient. The cells, displaying persistent random walk in the absence of FBS gradient, resort to chemotaxis on sensing FBS concentration. These results, when compared to similarly acquired ones but in the absence of the chemoattractant, confirm the capability of the simulation platform to capture such behaviors. The time interval between snapshots (left to right) is 1 hour. The final frame captures cell distribution at the end of 25 (physical) hours.

A virtual Insall chamber was constructed, based on the exact dimensions and specifications of the experimental setup, as obtained directly via private communication [62], [63] with the developers. To ensure consistency between the simulation and experiment, diffusion of FBS was modeled in the half of chamber containing the 0.5 mm bridge as shown in Figure 6. The geometry was discretized using structured grids (approximately 150,000 cells ensured grid independence). The diffusivity of FBS considered in the model was derived from the *Svedberg* [64] equation and calculated to be 8.705×10^−11^ m^2^/s. Virtual MV3 melanoma cells were modeled as spheres chemotacting at a speed of 8 µm/hr after sensing a critical FBS concentration (10% of the initial FBS concentration in the outer well). In the absence of the gradient, or in case the local gradient <10% FBS, cells migrated by displaying the persistent random walk. Cellular migration coupled to FBS transport in the virtual *Insall* chamber was modeled for a period of 25 physical hours. As a control, MV3 melanoma cell migration was modeled in the absence of FBS. Statistical significance was determined by conducting paired two-tailed test, where *p*<0.05 was interpreted as significant.

## Results

The integrated platform was used to create virtual bioreactors seeded with cells. Figure 2 shows the oxygen concentration contour plot as well as cell distribution inside one of the two different types of bioreactors we tested, at different time instances. The bioreactor is a rectangular prism in shape with two ports: one serving as an inlet (top left) and the other as an outlet (bottom right). Initially, the bioreactor was seeded with one hundred cells. The figures capture the interplay between cell population dynamics and the overall mass transport at various time steps – the final step was recorded at 4 physical days. The migration of cells from the relatively deoxygenated region (bottom right) of the bioreactor to top left can be observed. The dynamic nature of the relationship is best evident by the change in oxygen concentration close to the inlet port – it decreases in intensity in the subsequent time frames, corresponding to a decrease in concentration due to cellular proliferation and resulting increased consumption. Cell division in the region close to the inlet port was initially higher as compared to the rest of the reactor. This propensity of cells to divide closer to the inlet port where oxygen concentration is relatively high is behavior one would normally expect in reality. It must be stressed that this feature was not explicitly coded in the model but, it seems, emerged from the integration of the rule-set with underlying transport phenomena. Widespread proliferation is observed towards the end of the simulation as oxygen concentration exceeds the threshold value throughout the bioreactor. An interesting observation remains the preference the cells show in aligning themselves to the contour curves; thus resulting in an emergent banded distribution pattern – most evident in the second, third and fourth time frames.

Figure 3 shows the temporal evolution of cell population and nutrient concentration in a bioreactor (same as Figure 2) but with a medium inlet velocity (0.01 m/s), an order of magnitude higher than Case 1. As was the case in Figure 2, the cells tend to migrate away from the oxygen deficient region: chemotaxis. The top left region remains an area of high cell division during the initial stages. An important observation is the constant translation of the oxygen concentration contours, which can be better observed in Video S3. It is of interest to note that the banding pattern is significantly less pronounced in this case, and of a different shape than that observed in the previous virtual experiment most probably due to higher oxygen concentration relative to Case I. This behavior merits further investigation as it resonates, albeit modestly, with self-organization observed in biological systems. Questions such as: did lower oxygen availability in Case 1 cause the stressed cells to organize themselves in that manner; if so, can this be extrapolated to other cells, does such behavior lead to more efficient use of resources; what kind of structure would have evolved if cells were assigned more specific rules that govern colony formation, what would be the functionality of such structure; remain a matter of speculation until investigated more rigorously both computationally as well as empirically.

The probable cause behind the emergence of this distribution pattern is connected with the oxygen concentration, and related thresholds. The region close to the curve where patterning is observed is normoxic, whereas the region beyond the curve (closer to the outlet) is hypoxic. As a result cells migrate towards the curve under the influence of the concentration gradient and start behaving randomly as soon as they reach the normoxic region. In effect, the continuous supply of oxygen on one hand and the very random and unpredictable consumption on the other lead to a non-intuitive pattern formation, which spatially does not correspond to where the pure transport solution of the system would place the oxygen threshold iso-contour. Part of this emergent effect comes from the randomness inherent in cell (and agent) migration, but the most significant portion comes from the interplay of these two profoundly different mechanisms that such a hybrid methodology is well-positioned to capture. It must be noted that no matter which threshold is selected (within reasonable and biologically meaningful limits) the pattern formation is persistent in structure (of course varying slightly in exact position and formation) and thus clearly a feature of the coupled system. We found this very interesting and exciting emergent theme in many of the simulations we conducted. Such behavior can be exploited to create multiple mono-layers of defined thicknesses or bi-layers where cells towards the more deprived region of the bioreactor can act as an interface (as in a bone-cartilage hybrid structure).

**Figure 5:**
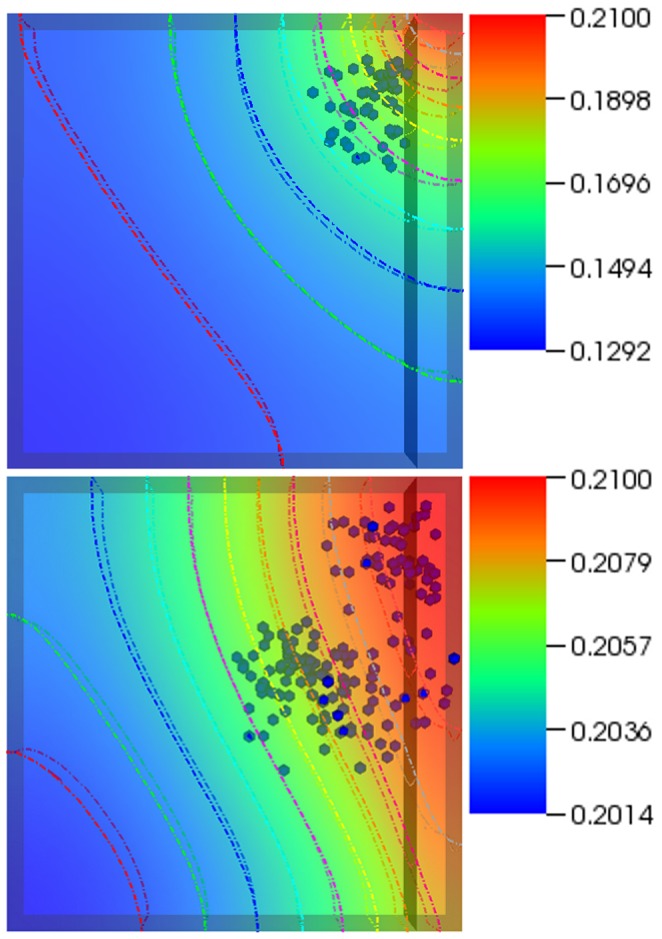
Different boundary conditions lead to different output. The figure shows temporal evolution of cell population and nutrient concentration in the same bioreactor set at different medium inlet velocities; 0.001 m/s (top) and 0.01 m/s (bottom). The bioreactor on the right ends up with considerably higher number of cells and a distinct growth pattern. This displays the dynamic nature of the system and the dependence of the spatiotemporal evolution of the system on processes such as chemotaxis and apoptosis. The frames were recorded at 5.5 days.

Figure 4 examines a different bioreactor setup. The top right end of the bioreactor serves as the inlet whereas the entire left as well as bottom ends of the bioreactor serve as the outlet. The medium inlet velocity in this case was 0.001 m/s. The bioreactor was randomly seeded with 5 cells. The simulation was run for a total of 6 physical days. By the second frame (17.5 hours), most of the cells in the deoxygenated region have died – a result of hypoxia-induced apoptosis. The cluster of cells formed by the final frame is a result of the *single* cell that was able to move and begin proliferating in the oxygen rich zone. When the medium inlet velocity was increased to 0.01 m/s (images not shown but supplementary video provided, Video S5), more cells survived which in turn aided in the colonization of the bioreactor. In comparison (Figure 5), the bioreactor with the higher inlet velocity by the final frame ends up with considerably higher number of cells and a distinct growth pattern (resembling the banding arrangement observed in the first case) – once again displaying not only the dynamic nature of the system but the dependence of the spatiotemporal evolution of the system on processes such as chemotaxis and apoptosis. It must be noted that, in some frames, a few cells still appear ‘red’ despite the local oxygen concentration being higher than the threshold. This is not connected with the simulation itself, but it is rather a visualization effect, utilized to highlight the hypoxic **history** of the relevant cells. The cells are no longer hypoxic and continue to grow as any other normoxic cell.

Figure 6 shows the evolution of FBS concentration gradient across the bridge and the cells’ migration response to the gradient over a period of 25 hours. Cell chemotaxis can be easily observed with the increase in FBS gradient towards the inner chamber. However, few cells that have not committed to chemotaxing can be observed at the end of the simulation on the left hand side of the bridge, but that is because these cells have not yet sensed the critical FBS concentration (10% of the initial concentration in the outer well). Qualitatively, the computational results (refer to Video S6) met expectations and were in very good agreement with the experiment conducted by Muinonen-Martin et al [60], where the melanoma cells were observed to migrate towards the outer well in a comparable manner. A highly significant statistical analysis (*p* = 1.14×10^−69^), also in agreement with its experimental counterpart, further supported the evidence for gradient directed migration of the MV3 melanoma cells inside the virtual chamber.

The importance of the cellular microenvironment to tissue development was hypothesized as early as 1817 but it took almost a century to confirm this hypothesis when certain regions of amphibian embryos were observed to direct the development of adjacent groups of cells to specific tissue types [41]. *Dynamic reciprocity* [40] takes this behavior a step further and suggests that there exists a dynamic bi-directional relationship between cells and their microenvironment, which is responsible for the overall development of the cellular system. Basically, “*the ECM affects the cell which in turn responds by synthetic and degradative processes causing the composition and the structure of ECM to change which in turn influences the cell and so forth”* [40]. In this paper, we presented a methodology that allows for the rigorous study of this interplay, expanded to include the local transport processes as a part of this synergistic interaction. After all, cellular proliferation *does* affect the local concentration gradients as well as flow profiles, thereby influencing the overall cell growth. In addition to devices such as bioreactors, the amended concept remains applicable to biological systems such as tumor, uterus or compromised tissue. The level of complexity associated with the process makes it quite difficult to be captured by numerical models. The lack of relevant biological data in addition to the complexity of such systems is a reason why comprehensive models for such systems have not been presented yet. However, investigators in the last decade have made significant progress in that direction as discussed in the Introduction.

The modeling platform presented in this manuscript was created keeping bioreactors in mind where the concentration profiles, shear stress and flow profiles etc. influenced initially by the geometry of the bioreactor and later by cellular proliferation play a crucial role in the synthesis of the autologous substitutes required for regenerative medicine. The modeling platform is composed of two working elements: continuum – transport phenomena capturing – and discrete – cellular behavior capturing – elements. Whereas the discrete layer helps the cells to detect the spatial information relevant for processes such as differentiation to occur [65], [66], the continuum layer helps the platform to model the dynamic transport processes that change continuously based on factors such as the number of cells, formation of ECM by the cell colonies or scaffold/ECM degradation. The biggest advantage of the platform, however, remains that it can capture emergent phenomena – a benefit extending from the agent-based side of the platform. As such, the platform can not only help explain non-intuitive observations but has the potential to reveal processes and mechanisms not expected to emerge *a priori*, like the cell band formations shown in the previous section.

A case in point is the 2D test case (video provided, Video S1) where the platform is able to capture the dynamic nature of the system. Secondly, cell alignment normal to the oxygen gradient was not a part of the rule-set attributed to the cells but emerged from it. A question that suggests itself at this point is whether such behavior can be manipulated to our advantage. Thirdly, in Figures 2 and 3 (and the corresponding videos, Videos S2 and S3) – especially in Figure 3–, the concentration contours change continuously, proving the effectiveness of the platform in capturing dynamic reciprocity as defined above. A physical system with no cells inside would not show such behavior after the flow becomes stationary, beyond initial transients.

The agent-based modality of the platform relies on biological rules and therefore the relevance and accuracy with which the framework can simulate a biological system will depend upon the validity of rules attributed to the agents. The simplest ways to achieve this include recourse to the data-mining paradigm or conducting statistical analyses on the data currently existing in the literature. Such methods can assist in evaluating critical parameters of a process; for example: the minimum concentration of a chemical that cells are sensitive to, the combination of growth factors that will direct stem cells to a particular lineage, the mechanical load cells must experience to differentiate into a particular lineage etc. These methods, despite their utility, might not by themselves, however, reveal the fundamental rules in biology – that endeavor rests with experimental biologists. We feel that targeted and quantitative experimental efforts, especially guided by mathematical tools such as the ones presented here, will assist in unearthing more rules that, in association with the underlying stochastic biophysicochemical processes, govern the dynamics of biological systems.

This modeling environment, which is under continuous expansion and development, can already capture chemotaxis and cell death (necrosis as well as apoptosis). Furthermore, it can be readily used to model features such as secretion of autocrine or paracrine molecules, production of metabolic waste products, cellular polarity etc. The platform can assist in conducting a quantitative as well as a qualitative analysis of how factors such as shear stress, pressure, and availability of nutrients/soluble factors can direct differentiation of cells in any physical system – and can be therefore utilized as a design tool in the bioreactor industry during the concept selection phase. The platform can also quantify the electro-magnetic fields that may exist in a system. This can help analyze further experiments such as the ones conducted by Zhao et al (2006) [67], who investigated the impact of electric signals of physiological strength in guiding cell migration – known as galvanotaxis – and wound healing. Simulations in this direction are currently underway in our lab. The next step involves factoring in the *de* novo secretion/formation (and possibly digestion/lysis) of extra-cellular matrix by cell colonies once they aggregate beyond a certain number and attributing to the ECM a unique set of properties based on the nature of cells secreting these fibers.

The results presented in this paper are in agreement with those from other models that were discussed above. Cheng et al (2009) [16] suggested that the hydrodynamic conditions affect not only the rate, but the pattern, of tissue growth as well, something we demonstrate in this paper: the supplementary videos show that migration speed, dependent on oxygen concentration, influences growth. Furthermore, one can easily observe (Figure 5) the way transport limitations affect spatial distribution of cells [16]. According to Cheng et al (2006) [15], by inducing a preferential migration direction oxygen concentration gradients influence cell migration. The platform captured that behavior evident in any of the figures and supplementary videos. Furthermore, the impact of bioreactor/scaffold geometry on cell proliferation can be also observed.

We have developed a modeling platform that captures the cell-level as well as population-level aspects of biological systems lending the platform the capability to capture the dynamism that is the signature of biology. Through the investigation reported here we successfully tested the research hypothesis that differences in initial and boundary conditions for the same volume can lead to non-identical development of a cellular system and that our platform is capable of capturing such variation. Furthermore, the platform was validated by simulating cellular chemotaxis and comparing the results with chemotaxis of MV3 melanoma cells under FBS concentration gradient in the Insall chamber [60]. Future developments include capturing cell colonization, ECM secretion by the colonies, and attributing the ECM with relevant biophysical information (porosity, spatial heterogeneity, diffusivity to certain molecules etc.).

### Conclusions

A novel way of simulating biological phenomena in bioreactors, especially dynamic reciprocity, was presented in this manuscript. The computational platform developed composed of two elements – agent-based and transport phenomena – is mature enough to model differentiation, chemotaxis and apoptosis in addition to cell proliferation, collision and persistent random walk. Most of the results discussed are in agreement with those obtained using models of similar purpose [15], [16], [21], [22]; in addition to showing behavior that may be emergent. The validated platform can be used as a design tool to test the impact of bioreactor geometries and experimental parameters on cell proliferation and differentiation in addition to supplementing the experimental techniques employed in gathering biological data.

## Supporting Information

Video S1 **2D test case displaying the dynamic nature of the system.** The video shows proliferating cells that are being fed via medium entering the system from left hand side of the construct. Continuous cell proliferation causes a drop in the concentration of nutrient medium inducing chemotaxis in the affected population. As the supply is unable to meet the demand, cells end up undergoing hypoxia-induced apoptosis.
(AVI)

Video S2 **Case 1 Video.** The video shows results of test case 1 (discussed in the paper).
(AVI)

Video S3 **Case 2 Video.** The video shows results of test case 2 (discussed in the paper). Notice especially the continuously changing concentration contours.
(AVI)

Video S4 **Case 3 Video.** The video shows results of test case 3 (discussed in the paper).
(AVI)

Video S5 **Case 4 Video.** The video shows results of test case 4. Similar to case 3 in its geometry, the medium flow rate used for this simulation is an order of magnitude higher in comparison. The difference in boundary conditions leads to distinct cell dynamics, which results in the formation of two different cell colonies.
(AVI)

Video S6 **Simulating chemotaxis in Insall Chamber.** The video shows results of the validation experiment conducted using the virtual Insall Chamber (discussed in the paper).
(AVI)
